# A searchable database of medical education objectives – creating a comparable gold standard

**DOI:** 10.1186/s12909-018-1136-z

**Published:** 2018-03-02

**Authors:** Sam Brooks, Namita Biala, Sage Arbor

**Affiliations:** 1 Marian University College of Medicine, 3200 Cold Spring Rd., Indianapolis, IN 36032 USA; 2 Department of Biological Sciences, Marian University, College of Osteopathic Medicine, 3200 Cold Spring Rd, Indianapolis, IN 46222 USA

**Keywords:** Medical, Objectives, Learning, Topics, Database, Integrated, Curriculum, Curricula

## Abstract

**Background:**

Medical school curricula strives to teach as much material as can be retained in a limited amount of time. A common “gold standard” resource used building curricula are medical objectives suggested by national societies. Unfortunately these objectives suffer from several functional limitations such as limited accessibility to society members, non-searchable formats (such as nested tables or pdf images), and inability to compare and search across societal objectives for redundancy or gaps. The shift towards integrated curriculums in medical school also highlights the need to access suggested content across classical discipline categories.

**Main body:**

We have codified recommendations from national societies in the United States for medical school objectives in a common tabular format and developed an open access database which can be searched across disciplines and societies. A front end website that allows for searching objectives by keyword while filtering on society or discipline was created. The objectives returned from the initial search can be subsearched by a second term. There is a large range in the format, age, breadth, quantity, and quality of objectives from different societies. Some unique disciplines have overlapping suggested content though most of the content does seem “binnable” by discipline. The choice of metadata for objectives from each given society was also very inconsistent.

**Conclusion:**

A free and searchable database of medical content to deliver during medical school has been developed with over 13,000 objectives from 18 societies and 22 disciplines at http://data.medobjectives.marian.edu/. The normalization of the different disciplines’ objectives into a common database allows a platform to standardize objectives moving forward. Future work could include adding user accounts to access the database, submission of new objectives, voting up and down suggested objectives, and adding “answers” mapped to objectives. Keyword tagging could allow import of content (e.g. PowerPoints) and outputting of suggested objectives, which would also allow comparison of curriculum across medical schools.

**Electronic supplementary material:**

The online version of this article (10.1186/s12909-018-1136-z) contains supplementary material, which is available to authorized users.

## Background

Medical school degree programs in different countries have durations that differ by multiple years but the final content of knowledge needed to be a doctor should be equivalent in depth and breadth. In the United States the scientific foundations to understand disease are generally taught in the first 2 years of medical school. Some of this information may have been presented to the students in undergraduate courses although specific prerequisites, such as biochemistry, vary by medical school in the United States. These first 2 years of medical school are usually the first time scientific knowledge is interwoven in a clinical context. Medical objectives recommended by national societies are often used for planning and following such a curriculum. However the accessibility and format of these recommended objectives often present barriers to their initial use and tracking. For example, objectives may only be accessible to society members behind a password protected website. In some cases the assembled objectives are only available as a hardcopy handout from a national meeting. The database assembled for this paper encountered both of these issues (see construction section). Curriculum at medical schools is assembled by a group of content experts with deliverable content often categorized by discipline since the basic science faculty have discipline specific training. The ability to search across disciplines is needed to develop a high caliber integrated curriculum, and aids in exposing both gaps and overlaps in curricular design or delivery.

Medical schools develop curricular mapping on their own, with their efficacy largely being judged based on post-graduation national exam board scores. In the United States there are currently two board tests USMLE (United States Medical Licensing Examination) and COMLEX (Comprehensive Osteopathic Medical Licensing Examination) required for allopathic and osteopathic medical students respectively, though there is significant crossover test taking as well. Both board review books (e.g. First Aid, Kaplan) and American national societies categorize the knowledge needed for these board tests by discipline. The frequency with which the societies update their suggested objective coverage varies widely, ranging from roughly every 3 to 20 years. The private companies update their review materials much more frequently, likely because the revisions are less exhaustive and there is a profit motive to do so. Other parts of the world have had similar efforts, the best example the authors are aware of concerning interschool objective mapping being the National Competence Based Catalogues of Learning Objectives for Undergraduate Medical Education (NKLM) and Dental Education (NKLZ) by the Association of Medical Faculties in Germany (MFT) which used as a starting point work done earlier by Switzerland, Canada, and the Netherlands [1]. The NKLM was not used in any way to make the current database, however it is worth comparing both the size and utility of the datasets. The NKLM had 1956 learning objectives in German compared to 13,121 objectives that are viewable in 103 languages in the current data set. The content of the database is derived from scientific societies based in the United States targeting medical students in the 4 year United States programs. In the United States medical school system there is usually a more rigorous structure during the first two largely didactic years, which is when content is tightly tied to objectives, and hence most of the database covers this medical education content. The latter 2 years of education in this system still has objectives covered in the database but they are less numerous and more broad. Therefore, while the database should prove useful in other systems the year of education the objectives will map to will be different. For example, in Europe medical school is a longer program (6 years) starting in undergraduate years and can begin directly out of high school, therefore many of the objectives in this database may be too detailed for early years of a European education, or could be split up over more time. The database does represent the final amount of knowledge that should be known/delivered before a student attains a medical degree. In addition, the database described in this paper represents the first time objectives suggested for coverage by national societies in the United States has been aggregated in one place and one format, and should provide a platform for continued submission of updated objectives for medical schools.

## Construction and content

Suggested medical school objectives were aggregated from disparate national societies in the United States of America (Table 1) [2–21]. The list of disciplines to cover were derived from discipline lists from the two types of medical schools in the United States, both allopathic [22] and osteopathic [23]. Of note osteopathic manipulation specific objectives, which would not be used in an allopathic school, were not included in the database by design since the purpose of the database was to create a resource that covered the agreed upon content necessary to master in order to become a doctor. In most cases a national society was found which represented a single discipline and had put forth material that it thought should be taught in the first 2 years of medical schools in the United States. There were two societies that covered more than one discipline in one document. The Association of Anatomy, Cell Biology, and Neurobiology Chairpersons (AACBNC) covered five disciplines: anatomy, cell biology, embryology, histology, and neuroscience [2]. The International Association of Medical Science Educators (IAMSE) covered both microbiology and immunology also in a single document [15]. On the other hand the Association of American Medical Colleges (AAMC) covered behavioral sciences [6] and clinical medicine [7], but at different times and in different documents. In addition, some societies, such as the Clerkship Directors in Internal Medicine and Society of General Internal Medicine (CDIMSHIM) [14], have content that is usually covered in years 3 and 4 of medical schools in the United States, during clinical rotations.

**Table 1:** Number of objectives in the database from each Society (sorted by discipline)

Discipline	Society Abbreviation	Society	Year
Anatomy	AACBNC [2]	Association of Anatomy, Cell Biology, and Neurobiology Chairpersons	1997
Anesthesia	SEA [3]	Society for Education of Anesthesia	2009
Behavioral Sciences	ADMSEP [4]	Association of Directors of Medical Student Education in Psychiatry	2007
Biochemistry	ABE [5]	Association of Biochemistry Educators	2012
Behavioral Sciences	AAMC [6]	Association of American Medical Colleges	2011
Cell Biology	AACBNC [2]	Association of Anatomy, Cell Biology, and Neurobiology Chairpersons	1997
Clinical Medicine	AAMC [7]	Association of American Medical Colleges	2008
Embryology	AACBNC [2]	Association of Anatomy, Cell Biology, and Neurobiology Chairpersons	1997
Family Medicine	STFM [8]	Society of Teachers of Family Medicine	2012
Genetics	APGMH [9]	Association of Professors of Human and Medical Genetics	2013
Genetics	ACMG [25]	American College of Medical Genetics	2011
Gerontology	SGE [12]	Studies in Geriatric Education	1986
Gerontology	AGHE [11]	Association for Gerontology in Higher Education	2008
Hematology	ASH [13]	American Society of Hematology	2014
Histology	AACBNC [2]	Association of Anatomy, Cell Biology, and Neurobiology Chairpersons	1997
Internal Medicine	CDIMSHIM [14]	Clerkship Directors in Internal Medicine and Society of General Internal Medicine	2006
Micro/Immuno	IAMSE [15]	International Association of Medical Science Educators	2009
Neuroscience	AACBNC [2]	Association of Anatomy, Cell Biology, and Neurobiology Chairpersons	1997
Obstetrics & Gynecology	APGO [16]	Association of Professors of Gynecology & Obstetrics	2009
Pathology	GRIPE [17]	Group for Research In Pathology Education	2005
Pediatrics	COMSEP [18]	Council on Medical Student Education in Pediatrics	2005
Pharmacology	ASPET [19]	American Society for Pharmacology and Experimental Therapeutics	2012
Physiology	APSACDP [20]	The American Physiological Society and the Association of Chairs of Departments of Physiology	2012
Radiology	AUR [21]	Association of University Radiologists	2014

Most of the objectives were available online in some format, usually pdf. The Association of Biochemistry Educators (ABE), formerly known as the Association of Biochemistry Course Directors (ABCD), had their objectives behind a password protected website and agreed to open up access for inclusion in this database as well as now making them freely accessible on their website [5]. A digital pdf copy had been created for the Association of Anatomy, Cell Biology, and Neurobiology Chairpersons (AACBNC), which at one point had floated around to various schools, but the objective text had been truncated in various text boxes. An original contributor to the objectives was eventually tracked down and one of two remaining hard copies was sent to be re-digitized and put in database format.

The objectives from disparate societies were tagged with largely non-overlapping metadata about each objective. An attempt was made to capture all metadata and include it in the database for retrieval. An excel template was created with 32 fields to allow others to populate with new objectives for import into the database moving forward (Additional file 1: Supplamental1–medicalObjectiveUpload.xls, and available on the website help page). The excel template has the fields listed in Table 2, which cover the types of metadata found with the objectives along with their frequency. Data was input into a MySQL database with a webpage set up for retrieval (http://data.medObjectives.marian.edu). A script to create the database with all data can be found in the supplemental data (Additional file 2: SupplementalDigitalContent2–medSchlObj-sqlCreate.txt). While the excel allows a flat file format to import new objectives, that data is split into multiple tables for querying in the database. The 3 currently used tables(columns) are:

1. objectives (see Table 2).
2. disciplines (discipline, displayName, inDB)
3. societies (name, abbrev, approvalLevel)

**Table 2:** Importable fields for future objectives (headings in Additional file 1)

Field Number(s)	Field	Current # of objectives with this data (%)
1	Author / Society Abbreviation	13,121 (100)
2	Year	13,121 (100)
3	Objective	13,121 (100)
4	Subheading 1	13,121 (100)
5	Subheading 2	12,787 (97.5)
6	Subheading 3	7555 (57.6)
7	Subheading 4	2984 (22.7)
8	Subheading 5	95 (0.7)
9	Subheading 6	0 (0)
10	Subheading 7	0 (0)
11–25	Keyword 1 thru 15	0 (0)
26	Notes	7102 (54.1)
27	Rank	1609 (12.2)
28	Hours	1214 (9.2)
29	Answer to objective	41 (0.3)
30	Discipline 1	13,121 (100)
31	Discipline 2	874 (6.7)
32	Discipline 3	0 (0)

Most fields require minimal space being stored as varchar < 256 with the exceptions in the objectives table being objective = varchar(2048), objectives notes = text, objective answer = text. These 3 fields allow longer objectives, and much longer notes and answers to the objectives. The database was developed and deployed on a HP ProLiant DL370 G6 blade server with 8 CPUs (Intel® Xeon® X5550 @ 2.67 GHz). A virtual machine on this server running Ubuntu 14.01 Linux houses the MySQL backend database, Apache server, as well as the PHP front end webpages.

When aggregating information, such as this medical objective database has done, it is ideal if none of the content has to be modified by third parties. However, since the objective database was designed to list objectives as at least one full sentence some decisions had to be made by the authors to make the objectives understandable. Examples of how this was done include converting tabular pharmacology objectives or explicitly restating content that was cross-referenced in societal objectives. The most troublesome objectives to incorporate were the pharmacology objectives from the American Society for Pharmacology and Experimental Therapeutics (ASPET) [19]. While there were many ASPET objectives that were clear in sentence form, there were often pharmacological substances whose names were only listed in nested tabular form. Therefore, many of these ASPET objectives had the pharmacological agents listed with the authors of this paper choosing to add the following constant phrase to end the objective: “describe the mechanism(s) of action, use(s), adverse effect(s), contraindication(s), and any relevant pharmacodynamic(s).” For example, because groups of drugs below were in two columns of a table labeled either “Nonselective Alpha Adrenergic Agonists” or “Selective Alpha_2_ Adrenergic Agonists”, the following two ASPET objectives were made from one table with different subheadings:

- “For the drugs DOPAMINE, EPINEPHRINE, NOREPINEPHRINE, phenylephrine, and pseudoephedrine as they pertain to synaptic and neuroeffector junctional sites - describe the mechanism(s) of action, use(s), adverse effect(s), contraindication(s), and any relevant pharmacodynamic(s).”
- “For the drugs BRIMONIDINE, CLONIDINE, and METHYLDOPA as they pertain to synaptic and neuroeffector junctional sites - describe the mechanism(s) of action, use(s), adverse effect(s), contraindication(s), and any relevant pharmacodynamic(s).”

In all cases authors chose to not modify the suggested objectives if possible, with the rare exception being fixing clear spelling errors. Student researchers compiled the database in a google doc flat file format, for ease of assembly, before import into the database. There was no data pre-processing/optimization before import into the database. Capitalization and punctuation was left unchanged, though search functionality was purposefully made not case sensitive.

All objectives have at a minimum an author (society), year, one subheading, and one discipline (Table 2). Fields that are listed which have zero occurrences were added to the database to allow for future growth when new objectives are added. Namely, up to 15 keywords are allowed which could allow for computational searching of objectives from non-human input sources (such as Power Points). An initial schema for the objective database allowed for unlimited keywords in a separate tall MySQL table, however it was decided limiting the keywords to 15 was acceptable to allow the ability to combine the tables in a flat format and allow future contributions via an excel file. Other non-existing classifications were considered such as: Blooms Taxonomy, clinical verse basic science knowledge, usual year of edification, and quality metric of the objective; however it was decided such fields would be better determined by the community in a future iteration of the database which could allow for user defined field creation. The most common form of metadata across societies was a top level subheading, which multiple objectives were grouped under. The notes section was also extensively used, often containing paragraph text from a heading above multiple objectives which applied to all objectives in a set.

## Utility and discussion

**Fig. 1:**
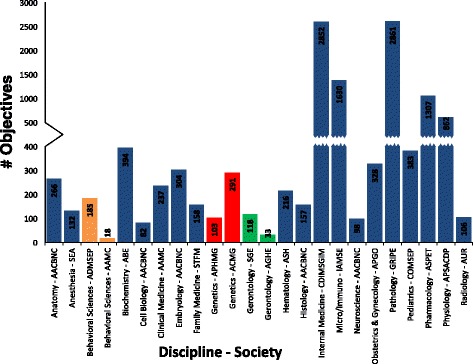
Number of Objectives by Discipline and Society. The number of suggested objectives per discipline varied by more than 100 fold (18 to 2861), and even within a single discipline varied up to 10 fold. Disciplines which only had objectives suggested by one society are shown by blue bars and are less likely to have redundant content suggested. Disciplines which had objectives listed redundantly in the database by more than one society are colored as follows: orange = behavioral sciences, red = genetics, and green = gerontology

For the first time a searchable database combining suggested medical objectives from societies in the United States across most disciplines has been created and made publically available. The search term must be in English, but there is a dropdown to convert all text to other languages which should make this tool useful outside of English speaking countries. This translation dropdown is available regardless of device use (desktop, tablet, phone) on the front search page, the results page, and the help page. Currently the pages can be translated into 103 languages. The number of objectives societies from disparate disciplines suggested be taught differed by roughly 100-fold with a minimum of 33 and maximum of 2852 objectives (Fig. 1). This content volume difference was largely due to a large spectrum in the specificity of objectives. In general, the basic science objectives seemed more discrete than the broader clinical objectives. For example, the 33 objectives from the Association for Gerontology in Higher Education were often broad such as “Compassion and understanding attitude on the part of the physician for care givers of the frail elderly and the difficulties they face” [24] whereas the Association of Biochemistry Educators had more numerous pointed objectives such as “Summarize the mechanism of DNA replication and why discontinuous synthesis is required” [5]. However a significant amount of the difference in the suggested number of objectives appeared due to societies differing in the depth with which they felt their content should be taught. Weighing the importance of the different disciplines’ content in order to assign medical students time is often hard to do when trying to compare siloed disciplines. The shared database provides transparency to disciplines’ requests of student’s time. A few disciplines were covered redundantly, by separate societies, and their suggested content also varied widely, at least 3–4 fold (Fig. 1). The two gerontology societies suggested 33 [24] and 118 [12] objectives, the two genetics societies suggested 103 [9] and 291 [25] objectives, and the two behavioral sciences societies suggested 18 and 185 objectives.

There are various use cases for such a medical objective database which can mostly be categorized by a single lecturers use versus a medical school using across their curriculum. An individual lecturer could use the database to discover gaps in their content as well as extraneous information they may be including. At the most simple level the database has already proven a useful tool for clinicians that lecture at Marian University but are not full time faculty. Since external clinicians often come in to give 1–4 lectures, but are not career educators, they are often not versed in how to make a “good” objective, and could use the database to quickly search words and then copy and paste objectives related to their lecture content in their PowerPoints. Having these pre-vetted objectives has also seemed to keep external lecturers “on message” and guide external clinicians in the design of their PowerPoints and lectures, because once the objective is listed in their second slide they need to make sure they cover that content in the remaining slides. Up to 100 objectives from a single search result can be copy and pasted into excel or a PowerPoint with two clicks (along with associated discipline, year, society, subheadings, and notes). A faculty member could quickly find objectives they likely should cover content for based on keyword searches but had not initially had in their PowerPoints, which often occurs when they are teaching about a disease state which has manifestations in a different discipline they did not understand well. A lecturers keyword search could also fail to retrieve many, or any, objectives which could hint that the content being delivered contains minutia that is likely beyond what the a new medical student needs to hear, or likely will be able to recall.

While the database has been “leaked” to a few colleagues at other schools during development, it has mainly been used at Marian University’s College of Osteopathic Medicine (COM), and mainly in the courses the developing faculty member (a biochemist) lectured in. Objectives have been searched for an initial foundational scientific knowledge course and biological system courses that followed: cardiac, pulmonary, renal, neuroscience, ear eyes nose and throat, dermatology, immunology, gastrointestinal, endocrine, metabolism, reproduction, and psychiatry. As a case example the psychiatry/behavioral course at Marian University was particularly heavy with external lecturers in the spring of 2017 and the database was used extensively. For that course all content (every sentence) from the psychiatry section of the First Aid board review book was mapped to a specific lecture in the course, and then much of that content had a corresponding objective from the database mapped to it. External lecturers were given all the content they were expected to deliver as well as suggested objectives. There were multiple changes made from the prior year, such as different lectures giving content, so a causal improvement due to the databases use cannot be made. Yet it seems worthy to note that in the first step board test (COMLEX step 1) taken shortly after this psychiatry/behavioral course the Marian University COM scores on the behavioral section improved from 460 to 602 in the early summer of 2016 and 2017 respectively (the national averages were 506 and 588 in 2016 and 2017 respectively). This represents a move from 0.25 standard deviations below the national average to 0.08 standard deviations above the national average and was that years greatest improvement in any discipline compared to the previous year’s class.

The medical objective database would likely yield greater results if adopted by an entire medical school. A medical school could decide they want to cover all the objectives in the database, or a subset thereof. Discipline specific societies likely often believe their content is more important to a medical degree than others would agree, so it is likely a subset of the database would be the desirable content to deliver in a medical education. Either way the database would then provide a checklist to go through and link which objectives are presented to students in each lecture. A first pass at this would expose “gaps” in the curriculum, i.e. objectives that should be covered but had not been assigned to a lecture. Redundancies would also be found, where multiple lecturers were repeating the same content. Redundancy is sometimes desired in medical school to hammer home certain content but, in the authors view, is more often an unintended consequence of each lecturer wanting to make their lecture self-contained enough to ensure a student can comprehend the content they are delivering.

## Conclusion

A free database has been designed which can search across disciplines and societies for medically relevant objectives (http://data.medObjectives.marian.edu). This single database provides transparency to compare between societies and disciplines, showing sometimes an order of magnitude difference in the number of objectives suggested to be covered during medical school for a given discipline (Fig. 1). Some of that discrepancy is due to the verbiage of disciplines covering a different level of scope on a topic, but there is also just a difference in coverage between suggested objectives. Nesting objectives under a hierarchical tree-of-knowledge, such as MeSH terms, could provide a metric for the scope of objective and content delivery.

The transparency yielded by having a single resource for medical objectives should help shed light on which disciplines have suggested less rigorous objectives. The aggregation of content not initially designed to be unified did present challenges. Most obvious were the nested pharmacological tabular objectives which did not have a clear way to translate themselves into didactic sentence format. An excel template (Additional file 1: Supplamental1–medicalObjectiveUpload.xls), also available on the help page of the website, provides a format for societies to add to or update their objectives. This standardized format to contribute future objectives should, if used, help normalize content across disciplines and possibly increase associated metadata content. For example, societies could choose to adopt metadata others have incorporated, such as suggested content hours per objective or including “answers” to objectives. Future work should include development of user accounts and addition of keywords, synonyms, and other datasets. It is envisioned that uploading of a school’s curriculum (e.g. a zip file of all Power Points), should allow automatic mapping to added keywords, resulting in a report (lecture by lecture) of which objectives should be covered. This would also allow for comparing of medical curriculum between schools, for example using board score data to measure the benefit or detriment of a curriculum spending time repeating content over new content delivery.

## Additional files

Additional file 1 An excel file with standardized format to submit new objectives to the database. (XLSX 25 kb)

Additional file 2 This file can be bulk uploaded into a blank mysql database to recreate this medical objective database with all content. (TXT 13173 kb)

## Abbreviations

AACBNC: Association of Anatomy Cell Biology and Neurobiology Chairpersons
AAMC: Association of American Medical Colleges
ABCD: Association of Biochemistry Course Directors
ABE: Association of Biochemistry Educators
ADMSEP: Association of Directors of Medical Student Education in Psychiatry
AGHE: Association for Gerontology in Higher Education
ASH: American Society of Hematology
ASPET: American Society for Pharmacology and Experimental Therapeutics
CDIMSHIM: Clerkship Directors in Internal Medicine and Society of General Internal Medicine
COM: College of Osteopathic Medicine
COMLEX: Comprehensive Osteopathic Medical Licensing Examination
CPU: Computer Processing Unit
IAMSE: International Association of Medical Science Educators
MFT: Association of Medical Faculties in Germany
NKLM: National Competence Based Catalogues of Learning Objectives for Undergraduate Medical Education
NKLZ: National Competence Based Catalogues of Learning Objectives for Dental Education
SEA: Society for Education of Anesthesia
SQL: Structured Query Language
USMLE: United States Medical Licensing Examination
